# The Shock of Intra-Abdominal Operations—Its Etiology, Prophylaxis and Treatment1Read by invitation before the Philadelphia County Medical Society, December 18, 1901.

**Published:** 1902-03

**Authors:** Lewis S. McMurtry

**Affiliations:** Louisville, Ky.


### SELECTION.

### The Shock of Intra-abdominal Operations—its Etiology, Prophylaxis and Treatment.[1]

By LEWIS S. McMURTRY, A. M., M. D., Louisville, Ky.

(*Proceedings of the Philadelphia County Medical Society, December, 1901.*)

BARRING exceptional and complicated cases, the causes of death after intraäbdominal operations are three: shock, hemorrhage and septic infection. The discovery of the relations of microörganisms to septic processes and the perfection of the modern aseptic surgical technique have practically overcome the last of these, so that in the hands of the modern abdominal surgeon it may be eliminated from the list. With the care given to hemostasis, in which the Trendelenburg position is a valuable aid, immediate and post-operative hemorrhage should be a rare cause of death. At any rate our knowledge of its serious import and our means for its control have reached a degree of accuracy and efficiency near to perfection. It is quite different, however, in relation to shock, which is still a potent factor of mortality, and regarding the nature, etiology, and pathology, of which we know little more now than when the subject was treated by Travers and Pirogoff. Clinical observation and experimental investigation have yielded only meager results in adding to our knowledge of the nature and mechanism of traumatic shock. This is a common complication of wounds and injuries, and must remain a subject of solicitous interest to the general and special surgeon until its nature, etiology, prevention, and therapy are more clearly comprehended.

Shock is an inhibition of the vital functions, marked by vasomotor paralysis. Its manifestations are through the nervous

1. Read by invitation before the Philadelphia County Medical Society, December 18, 1901.

system, and these relate especially to depressed and impaired action of the circulatory organs. It is evidently a neurosis, in which there is altered function of the nerve centers, with profound disturbance in the sympathetic nervous system. It varies in intensity from an evanescent and slight disturbance of nervous equilibrium to profound general depression and rapid dissolution. It is characterised by muscular relaxation, diminished cardiac force, lessened arterial tension, feeble respiration, arrest of glandular activity, and mental lethargy often verging into delirium. The symptoms of shock, when pronounced, present a striking clinical picture. The advent may be sudden, and the symptoms are those of most profound general depression. The pulse becomes rapid and feeble, the surface of the body is pallid and bathed in cold perspiration, the lips bloodless, the features pinched, and eyelids drooping. The respiration is feeble and irregular, and the temperature subnormal. The special senses are blunted, the mind lethargic and verging into unconsciousness. The secretory and excretory functions are in abeyance. These symptoms in the aggregate are usually of short duration. If they become more intense they speedily end in death; if the recuperative powers of the system triumph, the pulse and respiration improve, the heart acts with improved force and rhythm, the color returns to the skin, normal temperature is restored, the features regain normal appearance, and the mind resumes its sway.

In abdominal and pelvic surgery shock occurs during or immediately after operation. The terms secondary and delayed shock have tended to confusion in the study of this subject. Undoubtedly hemorrhage, acute sepsis, and fat embolism have been misinterpreted in this connection. These complications of wounds and injuries were formerly classified as delayed or secondary shock, terms most misleading. Modern surgeons no longer recognise such conditions as delayed or secondary shock.

In abdominal operations it is important to differentiate shock from chloroform asphyxia. In the latter condition the depression is sudden, with no warning symptoms; the pupils dilate; the respirations become irregular and cease; the pulse irregular, then ceases to beat at the wrist. When the anesthetic is discontinued, the patient suspended, an artificial respiration is induced, the pulse returns, and the alarming symptoms disappear. The less rapid accession of alarming symptoms and the lack of response to resuscitative treatment in shock will afford a basis of differentiation.

Sudden collapse from hemorrhage must also be differentiated from the nervous depression of shock. As a rule, the depression known as shock gradually appears during the late steps of the operation; the collapse of hemorrhage is immediate, and an expert anesthetiser will readily discriminate between the two conditions. The acute anemia of concealed and persistent hemorrhage is less readily differentiated from shock than the active hemorrhage just mentioned. Immediately after operation, when the abdomen has been closed and the patient placed in bed, hemorrhage will present many symptoms in common with shock. Differentiation here is most difficult. Indeed, many deaths resulting from concealed hemorrhage are attributed to shock. The pulse of hemorrhage is characterised by increasing frequency and diminishing force and volume. The face in hemorrhage has an anxious expression, but intelligent, the respiration is quick and sighing, and there is restlessness. The most difficult cases for diagnosis are those in which shock and hemorrhage jointly produce the depression. Should the symptoms of shock recur after the patient has partially rallied the existence of hemorrhage is almost assured. Such cases are illustrations of so-called secondary shock.

Acute septic infection may be confused with shock. These cases were quite common in abdominal surgery before the era of asepsis and during the period of imperfect asepsis. With a subnormal surface temperature the thermometer in the rectum or vagina will record a marked elevation above normal. The existence of conditions of possible sepsis will aid in the differentiation.

Emboli of various kinds may produce symptoms analogous to shock. Warren has called attention to the emboli of fat which occur in acute suppurations in fatty tissues, and which accumulate in the lungs, producing alarming symptoms and death.

The post-mortem disclosures in cases of death from shock, aside from the evidences of marked vascular disturbance, are purely negative. Shock must be regarded as a neurosis from a pathological standpoint.

In the etiology of shock individual susceptibility plays an important part. The resistance to injury in the animal creation is in proportion to the development of the nervous system. The lowest in the scale of development possesses the greatest resistance to injuries of all kinds. A highly endowed nervous organisation, whether acquired or hereditary, constitutes a predisposition to shock. The health of the individual is an important factor in the etiology of shock. Debilitating diseases, insufficient and indifferent food, dissipated habits, care and mental anxiety predispose to shock from slight injuries. In constitutions enfeebled by disease, especially when complicated by hemorrhage, operation will produce depression readily. This is especially manifest in cases of carcinoma. Repeated and long-continued hemorrhage likewise predisposes to shock, as illustrated in cases of uterine fibromata.

Dr. George W. Crile, of Cleveland, in a series of experimental investigations, has made a valuable contribution to the etiology of shock in abdominal operations.[1] These investigations are confirmed by practical experience. He found in a series of experiments upon dogs that shock is produced by opening the peritoneum; that simple exposure of the peritoneum to the atmosphere produced shock, the degree of shock varying inversely with the temperature of the air; the duration of operation and exposure was found to be an important factor of shock; manipulation of the peritoneum and enclosed organs induced symptoms of shock, increasing in intensity as the manipulation extended from the pelvis to the diaphragm; the same symptoms followed the disturbance of local splanchnic vasomotor areas and pressure upon the splanchnic veins, especially upon the vena cava.

These observations were made by means of the graphic method, whereby the alterations of the blood-pressure were recorded. Every experiment in the splanchnic area gave evidence that the dilatation of the vessels controlled by the splanchnic nerves was accompanied, *pari passu*, with the decline of pressure in the central and arterial circulatory apparatus. These nerves are vein nerves, and shock in operation on the splanchnic area is largely caused by the local disturbance of the vasomotor mechanism. The large splanchnic veins are engorged with blood in shock, and this we know is the result of impaired force of the heart's action; while the vasomotor mechanism plays an important rôle in shock, Crile's experiments show that this is not the whole cause. Clamping the thoracic aorta and splanchnic arteries did

1 The *American Gynecological and Obstetrical Journal* for March, 1898.

not prevent the symptoms and results of shock from injury to the intestines. It was also shown by Crile's observations that shock follows bloodless operations, thus disproving the assertion of some able clinicians that shock is but another name for hemorrhage. This important note, however, was confirmed by all experiments; "the less the insult to the tissue, the less the shock; the less the hemorrhage, the lighter the shock." The experimental investigations of Crile demonstrated throughout that hemorrhage, rude manipulation, peritoneal exposure, prolonged anesthesia, and loss of body heat are the most potent factors of shock after abdominal operations. These disclosures have long been recognised as clinical facts, and are confirmed by daily practical experience.

The prophylactic treatment of shock is most important. In all abdominal operations it should begin with the preparation of the patient for operation. While purgation, light diet, and increased elimination are essential features of preparatory treatment, such treatment should be very materially modified in the preparation of exhausted and debilitated patients as a preventive of shock. Active purgation and fasting predispose to shock. In patients exhausted by hemorrhage or debilitated by disease purgation should be omitted, and systematic feeding with bland concentrated liquid food up to within a few hours of the operation should be observed.

It was formerly quite the routine practice with many surgeons to administer morphine or alcohol, or both combined, preparatory to operation, in order to prevent shock. There is little either in experimental deduction or clinical experience to commend this practice. Crile found that preliminary administration of both these agents did not act favorably upon the phenomena of shock. Alcohol is still used for this purpose by some surgeons, being administered in the form of brandy or whiskey, diluted with water by enema prior to operation. While increasing the heart's force and vigor for a limited time, physiological investigation has shown that this period is succeeded by a period of depression which is conducive to shock. My own experience with this agent as a preventive of shock has not impressed me favorably. Opium is a depressor of vital power, and its physiological effects upon the nervous system are in the direction of promoting and increasing shock. These facts are recognised clinically by abdominal surgeons. Normal saline solution by enema preparatory to operation is an agent of positive value.

By increasing the venous pressure the output of the heart is increased and the contractions are stronger.

Both clinical experience and physiological experimentation attest the value of strychnia as a cardiac and vascular tonic, and therefore it is deservedly esteemed in practice as an agent of positive value in the prevention of shock. It should be administered in doses of 1-40 grain every six or eight hours for several days prior to operation in all cases wherein shock is apprehended, and 1-30 grain should be given hypodermatically immediately preceding anesthesia.

From what has already been stated as to the reduction of body temperature in the mechanism of shock, it is apparent that the temperature of the operating-room and the conserving of body heat by warm clothing should receive consideration in the prevention of shock. The temperature of the operating-room should be 80° F. the patient should be well wrapped in blankets, and bags of hot water placed within the folds of the blankets alongside the trunk and lower extremities. Contact of the skin with glass, tin, and other conductors of heat should be avoided.

It would be difficult to exaggerate the importance of care and skill in the administration of anesthetics in relation to the prophylaxis of shock. Sulphuric ether is not so pronounced in its depressing effects upon the heart and vasomotor system as chloroform, and requires less care in its administration. Not only the amount, but the method of administering anesthetics is important. The least quantity that will produce and maintain moderate surgical anesthesia, and the withdrawal of the drug at the earliest practicable moment in the final steps of the operation are essential points to be observed. Profound or prolonged anesthesia is per se a potent factor of shock.

Of all the causative factors of shock no one is so pronounced and potent as hemorrhage. In patients debilitated by disease and reduced by hemorrhage, it is wonderful how slight a hemorrhage during operation will depress the patient's vitality. The effect upon the heart is immediate. It is this clinical fact which has led some able surgeons to declare that shock is but another name for hemorrhage. From the beginning to the end of the operative procedure this fact should be constantly in the operator's mind. All active bleeding, both arterial and venous, should be arrested by the application of clamp forceps. While manipulating within the abdomen and deep pelvis, mechanical interference with large venous trunks should be avoided. Oozing surfaces should be packed with gauze pads wrung out of hot water, and constant attention devoted throughout the operation to arrest bleeding.

The experiments of Crile have demonstrated that a marked degree of shock is produced simply by opening the peritoneum and exposing the surface of that membrane to the atmosphere. Hence the shortest incision compatible with easy access and manipulation should be preferred. During the progress of the operation the visceral surface of the peritoneum should be protected with hot gauze pads, which will exclude atmospheric contact, preserve body-heat, and prevent surface evaporation.

The insult to the tissues, otherwise rude manipulation, is a potent and common cause of shock in abdominal operations. I know no one of the causes of shock which is so constantly disregarded as this. In no class of operations is a gentle touch more essential. Even when force must be applied, as in the separation of adhesions and enucleation of tumors, that force should be applied with due regard to the sensitive natures of the structures involved. Rude manipulation always shocks the patient, and much handling and stretching and twisting of structures covered by peritoneum can be avoided by care and manipulative skill—a skill to be cultivated by every surgeon who does this class of operations.

The duration of an operation bears a most conspicuous causative relation to shock. Long-continued anesthesia, even carefully administered, of itself will produce shock. The element of time in relation to anesthesia, to the exposed field and to manipulative irritation, is of the greatest moment in this connection. Hence, the mental concentration of the operator,—his command of technical detail and manipulative skill,—must play an important rôle in the prevention of shock.

*Treatment.*—The symptomatic indications for treatment in shock are to facilitate the cerebral circulation by position, using the Trendelenburg position on the operating-table and elevating the foot of the bed after operation. The heart must be strengthened by strychnine administered hypodermatically. Intravenous infusion of normal saline solution is indicated for reasons already given. The application of heat to the surface and the conservation of body-heat in every possible way are essential parts of treatment. Atropin is an agent of undoubted value in strengthening the heart. As the respiration is shallow and the blood insufficiently furnished with oxygen, artificial respiration and the administration of oxygen gas are indicated. Diffusible stimulants per enema (brandy or whiskey, f ij; warm water, ℥iv.) should be administered. In the effort to meet the issues presented, there is a constant temptation to repeat medicines in large doses too frequently.

